# The London Practice of Medicine and Surgery

**Published:** 1858-12

**Authors:** 


*The London Practice of Medicine and Surgery.—The Metropolitan Free Hospital.—Polycystic Ovarian Tumor—Ovariotomy—Recovery.* Under the care of Mr. HUTCHINSON.

We briefly mentioned a month ago two cases in which, on the same day, ovariotomy had been resorted to at the Metropolitan Free Hospital, and promised at a future opportunity to inform our readers of their result. We are now able to fulfil our pledge. In each instance the notes of the case have been supplied by the operator.

"On August 23, I met Mr. Tomlins, of Camden Town, in consultation in the case of Mrs. B., who had for more than a year been the subject of ovarian dropsy. She was a pale emaciated woman, aged 39, and had been reduced by the disease to a state of extreme debility.

For six weeks past she had not left her bed, and from the rapidity with which the emaciation, etc had advanced, it was plain that her case was not one in which, without interference, life would be long protracted. The tumor was a large one, filling to distension the whole abdomen, and giving the apperance of the full period of pregnancy. It was irregular in outline, and presented bossy projections in parts, some of which were very firm to the touch. Fluctuation was evident in most parts, and as a wave could easily be perceived on one side on percussing the other, it was probable that its front and larger portion consisted of a simple cyst. In the right loin the percussion note over a large space was clear and tympanitic, while in the left it was perfectly dull. The emaciation of the patient made the investigation of this sign very easy, and there could therefore be little or no doubt as to the side on which the disease had originated. All the pain which had been experienced was also referred to the left side. Her spirits were good, appetite fair, and the pulse, although very small, did not average more than eighty. Her aspect very nearly approached that indicative of malignant disease, but there was no history of cancer having ever showed itself in her family. Mrs. B. had been married several years, and was the mother of one child, now four years old. The catamenia since her child was weaned had been regular up to the present date. About a year ago she had noticed herself getting large in body, and her friends had suggested that she was pregnant. As her breasts were, however, wasting, and the monthly periods were regularly observed, she was herself unable to accept that explanation; and some doubt being felt, Dr. Murphy was called in, who at once informed her of the true nature of her disease. In conjunction with Dr. Murphy, Mr. Tomlins twice performed paracentesis, on the first occasion two months, and on the second, one month prior to my seeing her. The propriety of attempting extirpation of the cyst had been freely discussed, and both by her friends and by Mr. Tomlins, she had been strongly dissuaded from it. Dr. Murphy, on the contrary, had, I was informed, expressed an opinion favorable to its performance. It was a case, indeed, in which great difference of opinion might be allowably felt even among advocates of that operation. Her cachexia and debility, and the evidently solid character of the tumor, were far from encouraging circumstances. On the other hand, the rapid course which the disease was pursuing, made it evident that even should the operation end fatally, it would shorten her life by but a few weeks. Iodine injections were, of course, wholly out of the question. She was herself exceedingly anxious for the radical measure; and as this resolution had been come to in spite of an oft-repeated statement of its extreme danger, I felt myself warranted, after careful consideration of the various features of the case, in offering to undertake it. There was no time to be lost, and, accordingly, as it had been determined that she should come into the hospital, we admitted her at once.

"The operation was performed on August 30 (a very hot day,) and I was most efficiently assisted during it by my colleagues, Messrs. Chance and Childs. Before giving chloroform, a two grain opium pill was administered, and washed down by a glass of wine. The incision was commenced just below the navel. A considerable quantity of serous fluid escaped on opening the peritoneal cavity. As soon as the cyst was exposed, a very large trocar, the canula of which was supplied with a flexible tube, was introduced, and in about a minute the whole of its contents were evacuated. Extensive adhesions existed both in front and at the sides; but they were nowhere so strong but that by a little careful traction (supporting the peritoneum with the other hand.) I could tear them through. No cutting instrument was used for their division. The tumor, a very large irregular mass, having been lifted from the abdomen, its pedicle was found to be thick, short and vascular. It was secured in four portions by transfixing with strong hempen cord and around the whole was subsequently placed a single strong whipcord ligature. The latter was employed both as an additional safeguard against hæmorrhage and in order to permit of the more secure attachment of the end of the pedicle in the abominal incision. Some Some little dragging was unavoidable in the bringing the pedicle out externally; but as the abdominal walls were now flaccid, and admitted of being depressed backwards, it was not great. I stiched it with wire sutures firmly in place at the lowest part of the incision. The blood and fluid which had escaped into the abdominal cavity having next been taken up with warm flannel as efficiently as due attention to the value of time would permit, the incision was closed by deep wire sutures and long strips of plaister. The flannel bandage having been adjusted, the patient was removed to bed. Her pulse throughout the operation had been very fair, and it improved markedly as consciousness returned.

"Respecting her subsequent progress but little need be said, since, excepting so far as retarded by her very feeble condition, it was an unimpeded recovery. For the first six days she was supported almost solely by enemata; for although she never had any distress vomiting, her state of nausea and easily excited sickness forbade the giving of food by the mouth. During this period her pulse was rarely much less than 130, and her tongue, although quite clean readily became dry. The catheter was used twice daily for three days. As no pain was complained of, but little opium was given. Wine and brandy were freely employed, though not in any unusual quantities. The ligatures came away on the seventh day, and the wire sutures were removed on the eighth. Excepting the lower part, where the sloughing end of the pedicle prevented adhesion, the whole of the wound healed by first intention· At present the patient has an excellent appetite, is up daily, and will shortly leave the Hospital. A bedsore which showed itself a few days after the operation, and which had been undoubtedly caused by the long restriction to one position before its performance, has now nearly healed."

*Polycystic Ovarian tumor—Attempted Ovariotomy—Death From Peritonitis—Autopsy.* (Under the care of Dr. Ramskill and Mr. Hutchinson.)

The following narrative, like the preceeding, has been furnished by Mr. Hutchinson :—

" Mrs. E. aged 28, was admitted under Dr. Ramskill's care on April 8. Her abdomen was greatly distended, the disease dating from the preceding November. She had been treated prior to her admission for ascites, and had taken diuretics and Mercurials. Anteriorly the whole abdomen was perfectly dull on percussion, and fluctuation was easily felt and freely transmitted from part to part. The discovery that while the left loin was dull to percussion, the right was tympanitic, changed the diagnosis, and the removal by paracentesis of two bucketsful of fluid, of thick consistence and dark brown color, placed the opinion beyond doubt. After tapping, the abdomen subsided to a normal size, but the cyst could not be felt distinctly, while it was quite clear that a solid mass remained attached to the abdominal wall on the right aide, not far below the margin of the liver.

" The tumor rapidly refilled again, and much perplexity subsequently arose in determining to which side it belonged. The clearness of percussion in the right loin was not constant, while on the left side there was usually an extensive area over which the note was very superficially tympanitic, and which, although it never quite reached the loin, often encroached very closely on it.

" On June 19, July 24, and August 12, paracentesis was repeated. The fluid differed much in appearance on the several occasions, being on the second occasion like thin pus, and subsequently resembling gruel. On the two latter occasions the cyst was only partially emptied. The woman's health during this time had not given way materially. It had been determined on account of the known existence of extensive adhesions, not to attempt extirpation, and of this conclusion she had been informed. The day was fixed for her to leave the Hospital, and be transferred to the poor-house as a case not admitting of remedy. At her own most urgent wish, and on the repeated representation that she had rather encounter any amount of danger than remain as she was, provided there were the remotest chance of cure, I very reluctantly altered this decision, and consented to make an exploratory attempt.

"August 30—*The Operation.*—The abdomen was very large, sounded dull on percussion in its lower two-thirds and tympanitic in its upper third. The right loin on percussion on the operating table was ascertained to have a very clear resonance, while the left was quite dull. An opium pill, (gr. ii.) and a glass of wine, were given before the administration of the chloroform. On laying bare the cyst by an incision of about four inches long, I found it most firmly adherent in all parts. By a little patience, however, I succeeded in detaching the adhesions on the left side until the hand might be introduced beyond the knuckles between the abdominal wall and the cyst, and subsequently accomplished as much on the right side, without, however, entering the peritoneal cavity anywhere. This, however, encouraged me sufficiently to induce me to use the trocar, in the hope that after the withdrawal of the fluid, I might be able to proceed further. Only about half a pailfull of fluid escaped, and the abdomen was not much diminished in size. This puzzled us, and withdrawing the canula I slit up the cyst itself to an extent equal with that of the first incision in order to examine its interior. The hand now freely entered into the cavity of a large collapsed sac, the lining membrane of which was felt to be gritty from calcareous deposit in many places. Numerous small pedunculated secondary cysts projected into it, and the convolutions of the intestines were readily felt inverting, and deriving a reflected covering from it.—But what seemed to settle the question of any further attempt at removal was the finding high up above the umbilicus, depending into the upper part of the cyst, what felt exactly like the free edge of the liver. To this the cyst was firmly fixed, as if reflected over its whole surface.—My colleagues, Messrs. Chance and Childs, both of whom at my request introduced their hands, received the same impression as I had done as to the condition of parts. Without loss of time in any more detailed examination, I at once abandoned the operation, and closed the wound.

"The shock of the operation had been but little felt, and during the first twenty-four hours the patient's state was better than could have been expected. On the next day, however, most troublesome sickness set in, and scarcely left during the whole of the thirteen days that sl e subsequently lived. During that time I believe she scarcely slept, and her sufferings, despite the freest use of opium, were indeed extreme.—The wound did not heal, and most offensive glairy pus was discharged from the interior of the cyst. On the seventh day I introduced a trocar in the left side, where there was an extensive area of dulness on percussion, with fluctuation, in ordor to diminish the size of the abdomen, now greatly distended with flatus. Nearly a pailful of rather turbid ascitic serum was evacuated, and much relief afforded. On the tenth day erysipelas supervened, and spread rapidly over the whole trunk.—The pulse had throughout been rapid, and rather sharp, and the tongue dry and brown. To allay the distressing irritability of the stomach everything was tried that was thought likely, but without any result.—Death from exhaustion took place on the thirteenth day.

"*Autopsy.*—The peritoneal cavity contained more than a gallon of turbid serum mixed with flakes of lymph. In the pelvic regions it was lined everywhere by a thick homogeneous layer of lymph, which was coherent enough to allow of its being peeled off. Beneath this it presented everywhere extreme punctate congestion. The coils of intestine were distended with gas, and the stomach was of a very large size.—The cyst was now collapsed, and its membrane, which was thick and tough, was thrown in folds. In parts where it had been detached it was almost gangrenous. With regard to its adhesions, I had the mortification of finding that I had accomplished a large part of the task of detachment. The tumor had no adhesions posteriorly, and the left side, had I proceeded half of an inch further in detaching them, my hand would have been free in the peritoneal cavity. In the upper part of the sac, depending into its interior, was a large solid mass, which when cut into showed a good example of the firmer form of colloid deposit. Behind this mass were numerous small secondary cysts. This projecting mass it was which we had mistaken for the edge of the liver, it being firmly fixed by adhesions to the abdominal wall a little below that viscus. The tumor had adhesions in the intestines, omentum, etc., of rather an extensive character, and some of them very firm; there were none, however, but what I succeeded in detaching without lacerating the viscera. It was developed from the right ovary, and immediately behind it, accounting for the clear percussion note which had been heard in the right loin, was the cæcum distended with air. The dulness in the left loin had been due to ascitic fluid, which had been, as it were, encysted on the left side by the extensive adhesions of the tumor. The liver was united to the diaphragm by adhesions of date evidently long prior to the operation.

"In reviewing this case by the light thrown upon it by the autopsy, the regret which I feel is not that I was induced to attempt the operation, but that I did not persevere to its completion. To have wholly detached the tumor would have been a tedious and difficult affair; but with patience and care I have little doubt but that it might have been accomplished."

*Polycystic Ovarian Tumor—Ovariotomy—Favorable Progress.* Under the care of Dr. STAVELEY KING and Mr. HUTCHINSON.

On Monday week Mr. Hutchinson performed ovariotomy in a third case, the details of which we shall duly record, when its result is known. The tumor was a very large one indeed, and had most extensive adhesions. It was, however, safely removed, and at the present date the patient is in a very promising condition. For the securing of the peduncle, the operator employed, in place of the ligature, a steel clamp, which acted admirably and saved much time. The pedicle was large and thick, and contained several large artiries. Its extremity, togethcr with the clamp, were secured outside the incision.

[*London Medical Times & Gazette.*]

